# Systematic Review and Evidence Integration for Literature-Based Environmental Health Science Assessments

**DOI:** 10.1289/ehp.1307972

**Published:** 2014-04-22

**Authors:** Andrew A. Rooney, Abee L. Boyles, Mary S. Wolfe, John R. Bucher, Kristina A. Thayer

**Affiliations:** Office of Health Assessment and Translation, Division of the National Toxicology Program, National Institute of Environmental Health Sciences, National Institutes of Health, Department of Health and Human Services, Research Triangle Park, North Carolina, USA

## Abstract

Background: Systematic-review methodologies provide objectivity and transparency to the process of collecting and synthesizing scientific evidence in reaching conclusions on specific research questions. There is increasing interest in applying these procedures to address environmental health questions.

Objectives: The goal was to develop a systematic-review framework to address environmental health questions by extending approaches developed for clinical medicine to handle the breadth of data relevant to environmental health sciences (e.g., human, animal, and mechanistic studies).

Methods: The Office of Health Assessment and Translation (OHAT) adapted guidance from authorities on systematic-review and sought advice during development of the OHAT Approach through consultation with technical experts in systematic review and human health assessments, as well as scientific advisory groups and the public. The method was refined by considering expert and public comments and through application to case studies.

Results and Discussion: Here we present a seven-step framework for systematic review and evidence integration for reaching hazard identification conclusions: 1) problem formulation and protocol development, 2) search for and select studies for inclusion, 3) extract data from studies, 4) assess the quality or risk of bias of individual studies, 5) rate the confidence in the body of evidence, 6) translate the confidence ratings into levels of evidence, and 7) integrate the information from different evidence streams (human, animal, and “other relevant data” including mechanistic or *in vitro* studies) to develop hazard identification conclusions.

Conclusion: The principles of systematic review can be successfully applied to environmental health questions to provide greater objectivity and transparency to the process of developing conclusions.

Citation: Rooney AA, Boyles AL, Wolfe MS, Bucher JR, Thayer KA. 2014. Systematic review and evidence integration for literature-based environmental health science assessments. Environ Health Perspect 122:711–718; http://dx.doi.org/10.1289/ehp.1307972

## Introduction

Systematic-review methodologies increase the objectivity and transparency in the process of collecting and synthesizing scientific evidence on specific questions. The product of a systematic review can then be used to inform decisions, reach conclusions, or identify research needs. There is increasing interest in applying the principles of systematic review to questions in environmental health [European Food Safety Authority (EFSA) 2010; National Research Council (NRC) 2011, 2013a; Rhomberg et al. 2013; Woodruff and Sutton 2011].

Although systematic-review methodologies are well established in clinical medicine to assess data for reaching health care recommendations [Agency for Healthcare Research and Quality (AHRQ) 2013; Guyatt et al. 2011a; Higgins and Green 2011; Viswanathan et al. 2012], these approaches are most developed for human clinical trials, and therefore, typically consider small data sets of similar study design in developing conclusions. Questions in environmental health require the evaluation of a broader range of relevant data including experimental animal and mechanistic studies as well as observational human studies. Also, there is a need to integrate data from multiple evidence streams (human, animal, and “other relevant data” including mechanistic or *in vitro* studies) in order to reach conclusions regarding potential health effects from exposure to substances in our environment.

The National Toxicology Program (NTP) Office of Health Assessment and Translation (OHAT) conducts literature-based evaluations to assess the evidence that environmental chemicals, physical substances, or mixtures (collectively referred to as “substances”) cause adverse health effects and provides opinions on whether these substances may be of concern given levels of current human exposure (Bucher et al. 2011). Building on a history of rigorous and objective scientific review, OHAT has been working to incorporate systematic-review procedures in its evaluations since 2011 through a process that has included adoption of current practice, as well as methods development (Birnbaum et al. 2013; NTP 2012a, 2012b, 2013e). Here we explain the framework developed by OHAT that uses procedures to integrate multiple evidence streams including observational human study findings, experimental animal toxicology results, and other relevant data in developing hazard identification conclusions or state-of-the-science evaluations regarding health effects from exposure to environmental substances. The seven-step framework outlines methods to increase transparency and consistency in the process, but it also presents opportunities to increase efficiencies in data management and data display that facilitate the process of reaching and communicating hazard identification conclusions.

## Methods

In 2011, OHAT began exploring systematic-review methodology as a means to enhance transparency and increase efficiency in summarizing and synthesizing findings from studies in its literature-based health assessments. OHAT used a multipronged strategy to develop the OHAT Approach, working with advisors to adapt and extend existing methods from clinical medicine and obtaining input from technical experts and the public on early drafts (see Supplemental Material, Table S1). The methods-development process is described in detail in Supplemental Material (“Process for developing the OHAT Approach,” pp. 2–7). In brief, OHAT reviewed guidance from authoritative systematic-review groups (AHRQ 2013; Guyatt et al. 2011a; Higgins and Green 2011) in developing an initial draft and sought additional advice through web-based discussions and consultation with technical experts, the NTP Executive Committee, the NTP Board of Scientific Counselors, and the public (NTP 2012a, 2012b, 2013b, 2013c, 2013d, 2013e). The resulting OHAT Approach has been refined based on the input received and through application to case studies.

## Results

**Figure 1:**
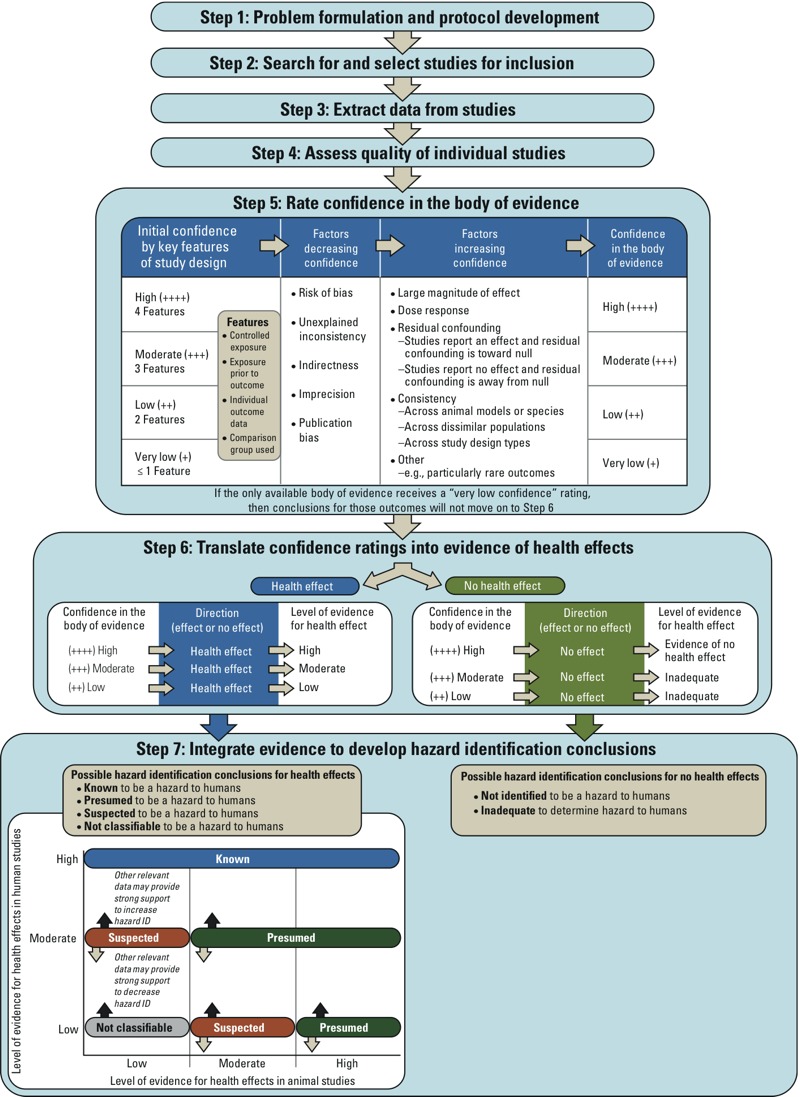
The OHAT Approach for systematic review and evidence integration for literature-based environmental health science assessments.

The OHAT framework is a flexible seven-step process (Figure 1) tailored to the complexity of the research question. It includes all of the recommended elements for conducting and reporting a systematic review [outlined in the PRISMA (Preferred Reporting Items for Systematic Reviews and Meta-Analyses) statement (Moher et al. 2009)]. The specific procedures for performance of each step are described in a detailed protocol developed for each evaluation (NTP 2013a, 2013f).

### Step 1: Problem Formulation and Protocol Development

Prior to conducting an evaluation, the scope and focus of the topic is defined through consultation with subject-matter experts. For OHAT, the objective is typically to identify a potential health hazard or assess the state of the science in order to identify research needs on topics of importance to environmental health. The objectives of the evaluation must be clearly stated, including the key questions to be addressed. The evaluation is structured to answer these key questions that guide the systematic-review process for the literature search, study selection, data extraction, and synthesis. The questions define the populations, exposures, comparators, outcomes, timings, and settings of interest (PECOTS) eligibility criteria for the evaluation (e.g., see discussion in AHRQ 2013). PECOTS is the environmental equivalent of AHRQ’s PICOTS expansion of the original PICO approach developed for clinical evaluations that focuses on interventions rather than exposures, and did not initially include timing or setting in the inclusion criteria (Whitlock et al. 2010).

A concept document (or brief proposal) and a specific, detailed protocol for OHAT evaluations are developed through an iterative process in which information is obtained by outreach to federal partners, technical experts, and the public and through consultation with the NTP Board of Scientific Counselors (NTP 2013g). Through this process, the protocol is developed *a priori*, and guidance in the protocol forms the basis for scientific judgments throughout the evaluation. However, it is important to acknowledge that the protocol can be modified to address unanticipated issues that might arise while conducting the review (e.g., see Food and Drug Administration 2010; Khan et al. 2001). Revisions to the protocol are documented and justified with notation of when in the process the revisions were made.

### Step 2: Search for and Select Studies for Inclusion

*Search for studies.* A comprehensive search of the primary scientific literature is performed. The search covers multiple databases (including, but not limited to, PubMed, TOXNET, Scopus, and Embase) with sufficient details of the search strategy documented in the protocol such that it could be reproduced. The protocol also lists the dates of the search, frequency of updates, and any limits placed on the search (e.g., language, date of publication). The protocol establishes requirements for consideration of data from meeting abstracts or other unpublished sources. If a study that may be critical to the evaluation has not been peer reviewed and the authors agree to make all study materials available, the NTP will have it peer reviewed by independent scientists with relevant expertise. The peer-review requirement assures that studies considered in the evaluation have been reviewed by subject-matter experts, and the information from this review would be available in step 4 when evaluating individual study quality.

*Select studies for inclusion.* All references identified in the search are screened for relevance to the key question(s) of the evaluation based on the PECOTS eligibility criteria established when formulating the problem in step 1. The protocol establishes criteria for including or excluding references based on, for example, applicable outcomes, relevant exposures, and types of studies. These criteria contain sufficient detail to develop an inclusion and exclusion checklist in order to limit the use of scientific judgment during the literature-selection process. If major limitations in a specific study type or design for addressing the question are known in advance (e.g., unreliable methods to assess exposure or health outcome), the basis for excluding those studies must be described *a priori* in the protocol.

The protocol also outlines the specific plans for reviewing studies for inclusion, resolving conflicts between reviewers, and documenting the reasons that studies were excluded. Two reviewers independently screen all references at the title and abstract level and resolve differences by reaching agreement through discussion. References that meet the inclusion criteria are retrieved for full text review, as are those with insufficient information to determine eligibility from just the title and abstract. Procedures for full text review are tailored to the scope of the review and follow procedures established in the protocol. Creating a flow diagram to show the number of references retrieved, duplicates removed, and studies excluded as references move through the screening process is one of several required elements for reporting based on the PRISMA statement (Liberati et al. 2009; Moher et al. 2009) that we have included in this framework.

### Step 3: Extract Data from Studies

Relevant data from individual studies selected for inclusion are extracted or copied from the publication to a database to facilitate critical evaluation of the results, including data summary and display using separate data collection forms for human, animal, and *in vitro* studies. For each study, one member of the evaluation team performs the data extraction, and quality assurance procedures are undertaken as specified in the protocol (e.g., review and confirmation by another team member). Following completion of an evaluation, the data extracted and summarized will be made publicly available in the NTP Chemical Effects in Biological Systems (CEBS) database (NTP 2014a).

### Step 4: Assess the Quality or Risk of Bias of Individual Studies

Despite the critical importance of assessing the credibility of individual studies when developing literature-based evaluations, the meaning of the term “quality” varies widely across the fields of systematic review, toxicology, and public health (see discussion in Viswanathan et al. 2012). Broadly defined, study quality includes *a*) reporting quality (how well or completely a study was reported); *b*) internal validity or risk of bias (how credible the findings are based on the design and apparent conduct of a study); and *c*) external validity or directness and applicability (how well a study addresses the topic under review) (see Cochrane Collaboration 2013 for detailed definitions). Study quality assessment tools that mix different aspects of study quality or provide a single summary score are discouraged (Balshem et al. 2011; Higgins and Green 2011; Liberati et al. 2009; Viswanathan et al. 2012).

The OHAT risk-of-bias tool adapts guidance from the AHRQ (Viswanathan et al. 2012). Individual risk-of-bias questions are designated as applicable only to certain types of study designs (e.g., human controlled trials, experimental animal studies, cohort studies, case–control studies, cross-sectional studies, case series or case reports), with a subset of the questions applying to each study design (Table 1).

**Table 1:** OHAT risk-of-bias questions.

Bias categories and questions	Applicable study designs
Selection bias
**Was administered dose or exposure level adequately randomized?** Randomization requires that each human subject or animal had an equal chance of being assigned to any study group, including controls (e.g., use of random number table or computer generated randomization).	ExA,^*a*^ HCT^*b*^
**Was allocation to study groups adequately concealed?** Allocation concealment requires that research personnel do not know which administered dose or exposure level is assigned at the start of a study. Human studies also require that allocation be concealed from human subjects prior to entering the study. *Note: *a*) a question under performance bias addresses blinding of personnel and human subjects to treatment during the study; *b*) a question under detection bias addresses blinding of outcome assessors.*	ExA, HCT
**Were the comparison groups appropriate? ** Comparison group appropriateness refers to having similar baseline characteristics between the groups aside from the exposures and outcomes under study.	Coh,^*c*^ CaC,^*d*^ CrS^*e*^
Confounding bias
**Did the study design or analysis account for important confounding and modifying variables? ** *Note: a parallel question under detection bias addresses reliability of the measurement of confounding variables.*	All^*f*^
**Did researchers adjust or control for other exposures that are anticipated to bias results?**	All
Performance bias
**Were experimental conditions identical across study groups?**	ExA
**Did researchers adhere to the study protocol?**	All
**Were the research personnel and human subjects blinded to the study group during the study? ** Blinding requires that study scientists do not know which administered dose or exposure level the human subject or animal is being given (i.e., study group). Human studies require blinding of the human subjects when possible.	ExA, HCT
Attrition/exclusion bias
**Were outcome data complete without attrition or exclusion from analysis? ** Attrition rates are required to be similar and uniformly low across groups with respect to withdrawal or exclusion from analysis.	ExA, HCT, Coh, CaC, CrS
Detection bias
**Were the outcome assessors blinded to study group or exposure level? ** Blinding requires that outcome assessors do not know the study group or exposure level of the human subject or animal when the outcome was assessed.	All
**Were confounding variables assessed consistently across groups using valid and reliable measures? ** Consistent application of valid, reliable, and sensitive methods of assessing important confounding or modifying variables is required across study groups. *Note: a parallel question under selection bias addresses whether design or analysis account for confounding.*	All
**Can we be confident in the exposure characterization? ** Confidence requires valid, reliable, and sensitive methods to measure exposure applied consistently across groups.	All
**Can we be confident in the outcome assessment? ** Confidence requires valid, reliable, and sensitive methods to assess the outcome and the methods should be applied consistently across groups.	All
Selective reporting bias
**Were all measured outcomes reported?**	All
Other
**Were there no other potential threats to internal validity (e.g., statistical methods were appropriate)? ** On a project-specific basis, additional questions for other potential threats to internal validity can be added and applied to study designs as appropriate.	Additional items as applicable by study design
The OHAT risk-of-bias questions are applied to evaluate the risk of bias of studies on an outcome basis. The study design types to which each risk-of-bias question applies are given in the right-hand column. Answering “yes” indicates lower risk of bias, whereas “no” indicates higher risk of bias for that question. Risk-of-bias ratings are developed by answering each applicable question with one of four options (definitely low, probably low, probably high, or definitely high risk of bias). Abbreviations: CaC, case–control; CaS, case series; Coh, prospective or retrospective cohort; CrS, cross-sectional; ExA, experimental animal; HCT, human controlled trial. ^***a***^ExA studies are controlled exposure studies; nonhuman animal observational studies could be evaluated using the design features of observational human studies such as CrS study design. ^***b***^HCTs are carried out in humans using a controlled exposure, including randomized controlled trials and non-randomized experimental studies. ^***c***^Coh studies include prospective studies that follow subjects free of disease over time or retrospective studies of subjects with prior information available. ^***d***^CaC studies enroll subjects based on their disease status and compare exposures across the groups. ^***e***^CrS studies are conducted at one point in time and include population surveys with individual data [e.g., National Health and Nutrition Examination Survey (NHANES)] and population surveys with aggregate data (i.e., air pollution exposure estimated by ZIP code). ^***f***^All applies to ExA, HCT, Coh, CaC, and CrS studies, as well as other study design types such as case reports or CaS studies that lack a comparison group within the study.	

Published tools do not address risk-of-bias criteria for animal studies because risk-of-bias tools, as with systematic-review methods in general, have been focused on guidelines for clinical medicine. OHAT evaluates risk of bias in experimental animal studies using criteria similar to those applied to human randomized controlled trials, because these study designs are similar in their ability to control timing and dose of exposure and to minimize the impact of confounding factors. Using the same set of questions for all study types, including experimental animal studies, allows for comparison of particular risk-of-bias issues across a body of evidence and facilitates comparison of the strengths and weaknesses of different bodies of evidence.

All references are independently assessed for risk of bias for each outcome of interest by two reviewers who answer all of the applicable questions with one of four options (definitely low, probably low, probably high, or definitely high risk of bias) (CLARITY Group at McMaster University 2013) following prespecified criteria detailed in the protocol. Before proceeding with the risk-of-bias assessment, OHAT recommends evaluating a small subset of studies as a “pilot” to clarify how the protocol-specific criteria will be applied through dialogue among subject matter experts and reviewers. During completion of the risk-of-bias assessment for the full set of studies, discrepancies between the reviewers are resolved by reaching agreement through discussion.

### Step 5: Rate the Confidence in the Body of Evidence

For each outcome, the confidence in the body of evidence is rated by considering the strengths and weaknesses of a collection of studies with similar study design features. Ratings reflect confidence that the study findings accurately reflect the true association between exposure and effect including aspects of external validity (or directness and applicability) for the studies. The OHAT method is based on the Grading of Recommendations Assessment, Development and Evaluation (GRADE) Working Group guidelines (GRADE 2014), which have been adopted by the Cochrane Collaboration (Schünemann et al. 2012) and AHRQ approaches (Balshem et al. 2011; Lohr 2012), which are conceptually very similar. The OHAT method uses four descriptors to indicate the level of confidence in the separate bodies of evidence (Table 2). In the context of identifying research needs, a conclusion of “high confidence” indicates that further research is very unlikely to change the confidence in the apparent relationship between exposure to the substance and the outcome. Conversely, a conclusion of “very low confidence” suggests that further research is very likely to impact confidence in the apparent relationship. Human and nonhuman animal data are considered separately throughout Steps 5 and 6. Conclusions developed in the subsequent steps of the approach are based on the evidence with the highest confidence.

**Table 2:** Confidence ratings in the bodies of evidence.

Confidence rating	Definition
High confidence (++++)	High confidence in the association between exposure to the substance and the outcome. The true effect is highly likely to be reflected in the apparent relationship.
Moderate confidence (+++)	Moderate confidence in the association between exposure to the substance and the outcome. The true effect may be reflected in the apparent relationship.
Low confidence (++)	Low confidence in the association between exposure to the substance and the outcome. The true effect may be different from the apparent relationship.
Very low confidence (+)	Very low confidence in the association between exposure to the substance and the outcome. The true effect is highly likely to be different from the apparent relationship.

For each outcome, studies are given an initial confidence rating that reflects the presence or absence of key study-design features (Figure 1, step 5). Then studies that have the same number of features are considered together as a group to begin the process of rating confidence in a body of evidence for that outcome. The initial rating of each group is downgraded for factors that decrease confidence and upgraded for factors that increase confidence in the results. Confidence across all studies with the same outcome is then assessed by considering the ratings for all groups of studies with that outcome, and the highest rating for that outcome moves forward.

Although confidence ratings for each outcome are developed for groups of studies, the number of studies constituting the group will vary, and in some cases this group may be represented by only one study. Therefore, it is worth noting that a single well-conducted study may provide evidence of toxicity or a health effect associated with exposure to the substance in question [e.g., see Germolec (2009) and Foster (2009) for explanations of the NTP levels of evidence for determination of “toxicity” for individual studies]. If a sufficient body of very similar studies is available, a quantitative meta-analysis may be completed to generate an overall estimate of effect, but this is not required. Finally, confidence conclusions are developed across multiple outcomes for those outcomes that are biologically related.

It is recognized that the scientific judgments involved in developing these confidence ratings are inherently subjective. A key advantage of the systematic-review process for this step and throughout an evaluation is that it provides a framework to document and justify the decisions made, and thereby provides for greater transparency in the scientific basis of judgments made in reaching conclusions.

*Initial confidence set by key features of study design for each outcome.* An initial confidence rating is determined by the ability of the study design to address causality as reflected in the confidence that exposure preceded and was associated with the outcome (Figure 1, step 5). This ability is reflected in the presence or absence of four key study-design features that determine initial confidence ratings, and studies are differentiated based on whether *a*) the exposure to the substance is controlled; *b*) the exposure assessment represents exposures occurring prior to development of the outcome; *c*) the outcome is assessed on the individual level (i.e., not population aggregate data); and *d*) a comparison or control group is used within the study. The first key feature, “controlled exposure,” reflects the ability of experimental studies in humans and animals to largely eliminate confounding by randomizing allocation of exposure. Therefore, these studies will usually have all four features and receive an initial rating of “high confidence.” Observational studies do not have controlled exposure and are differentiated by the presence or absence of the three remaining study-design features. For example, prospective cohort studies usually have all three remaining features and receive an initial rating of “moderate confidence,” whereas a case report may have only one key feature and receive an initial rating of “very low confidence” (see Supplemental Material, Table S2, for key features for standard study designs and discussion, pp. 9–11). The presence or absence of these study-design features capture and discriminate studies on an outcome-specific basis (e.g., experimental, prospective) but do not replace consideration of risk of bias elements or external validity in other steps.

*Downgrade confidence rating.* Five properties of the body of evidence (risk of bias, unexplained inconsistency, indirectness, imprecision, and publication bias) are considered to determine if the initial confidence rating should be downgraded (Figure 1, step 5). For each of the five properties, a judgment is made and documented regarding whether there are substantial issues that decrease the confidence rating in each aspect of the body of evidence for the outcome. Factors that would downgrade confidence by one versus two levels are specified in the protocol. The reasons for downgrading confidence may not fit neatly into a single property of the body of evidence. If the decision to downgrade is borderline for two properties, the body of evidence is downgraded once to account for both partial concerns. Similarly, the body of evidence is not downgraded twice for what is essentially the same limitation that could be considered applicable to more than one property of the body of evidence.

Risk of bias of the body of evidence. Risk-of-bias criteria were described in step 4 in which study-quality issues for individual studies are evaluated on an outcome-specific basis. In step 5, the previous risk-of-bias assessments for individual studies now serve as the basis for an overall risk-of-bias conclusion for the entire body of evidence. Downgrading for risk of bias should reflect the entire body of studies; therefore, the decision to downgrade should be applied conservatively. The decision to downgrade should be reserved for cases for which there is substantial risk of bias across most of the studies composing the body of evidence (Guyatt et al. 2011e).

Unexplained inconsistency. Inconsistency, or large variability in the magnitude or direction of estimates of effect across studies that cannot be explained, reduces confidence in the body of evidence. Large inconsistency across studies should be explored, preferably through *a priori* hypotheses that might explain the heterogeneity.

Indirectness. Indirectness can refer to external validity or indirect measures of the health outcome. Indirectness can lower confidence in the body of evidence when the population, exposure, or outcome(s) measured differs from the population, exposure, or outcome(s) that is of most interest. Concerns about directness could apply to the relationship between *a*) a measured outcome and a health effect (i.e., upstream biomarker of a health effect); *b*) the route of exposure and the typical human exposure; *c*) the study population and the population of interest (Guyatt et al. 2011c; Lohr 2012); *d*) the timing of the exposure relative to the appropriate biological window to affect the outcome; or *e*) the timing of outcome assessment and the length of time required after an exposure for development of the outcome (Viswanathan et al. 2012).

The administered dose or exposure level is not considered a factor under indirectness for developing a confidence rating for the purpose of hazard identification. Although exposure level is an important factor in considering the relevance of study findings to human health effects at known human exposure levels, in the OHAT evaluation process, this consideration occurs after hazard identification as part of reaching a “level of concern” conclusion (Jahnke et al. 2005; Medlin 2003; Shelby 2005; Twombly 1998). The accuracy of an exposure metric (e.g., market basket survey vs. individual blood levels of a substance) is also not considered a factor under indirectness, and the confidence in the exposure assessment is considered in the risk-of-bias evaluation of individual studies on an outcome basis in step 4.

Imprecision. Imprecision is the lack of certainty in an estimate of effect for a specific outcome. A precise estimate enables the evaluator to determine whether there is an effect (i.e., it is different from the comparison group). Confidence intervals for the estimates of effect provide the primary evidence used in considering the imprecision of the body of evidence (Guyatt et al. 2011b).

Publication bias. Publication bias is addressed specifically in rating the body of evidence, and selective reporting within a study is covered in the risk-of-bias criteria addressing these limitations (Guyatt et al. 2011d). Funnel plots provide a useful tool to visualize asymmetrical or symmetrical patterns of study results for assessing publication bias when there is a sufficient body of studies for a specific outcome (e.g., Ahmed et al. 2012). There is empirical evidence that studies with negative results (null findings for clinical trials) are less likely to be in the published literature (Hopewell et al. 2009). Negative studies may also be affected by “lag bias” or longer time to publication (Stern and Simes 1997); therefore, it is important to carefully consider data sets that are limited to few positive studies with small sample size that might indicate a lag time between early positive studies and lagging negative studies. Although some publication bias is expected, downgrading is reserved for cases in which serious concern for publication bias significantly decreases confidence in the body of evidence.

*Upgrade confidence rating.* Four properties of the body of evidence (large magnitude of effect, dose response, residual confounding increases confidence, and cross-species/population/study consistency) are considered to determine if the confidence rating should be upgraded (Figure 1, step 5). For each of the four properties, a judgment is made and documented regarding whether or not there are substantial factors that increase the confidence rating in the body of evidence for the outcome. As discussed above for downgrading, two borderline upgrades could be combined for one upgrade and the body should not be upgraded twice for essentially the same attribute. Factors that would upgrade confidence by one versus two levels are specified in the protocol.

Large magnitude of effect. A large magnitude of effect is defined as an observed effect that is sufficiently large so that it is unlikely to have occurred as a result of bias from potential confounding factors.

Dose response. A plausible dose–response relationship between the level of exposure and the outcome increases confidence in the result because it reduces concern that the result could be due to chance. In addition to considering dose response within a study with a range of exposure levels, consideration of multiple studies with varied exposure levels can contribute to an overall picture of the dose response. It is important to recognize that prior knowledge may lead to an expectation for a nonmonotonic dose response. Therefore, the plausibility of the observed biological response should be considered in evaluating the dose–response relationship.

Residual confounding increases confidence. This element refers to consideration of residual confounding, healthy worker effect, or effect modification that would bias the effect estimate toward the null. If a study reports an effect or association despite the presence of residual confounding that would diminish the association, confidence in the association is increased. This confounding can push in either direction; therefore, confidence in the results are increased when there is an indication that a body of evidence is potentially biased by factors counter to the observed effect.

Cross-species/population/study consistency. Three types of consistency in the body of evidence can increase confidence in the results: across animal studies (consistent results reported in multiple experimental animal models or species); across dissimilar populations [consistent results reported across populations (human or wildlife) that differ in factors such as time, location, and/or exposure]; and across study types (consistent results reported from studies with different design features).

Other. Additional factors specific to the topic being evaluated (e.g., particularly rare outcomes) may result in increasing a confidence rating. These other factors would be specified and defined in the protocol.

*Combine confidence conclusions for all study types and multiple outcomes.* Conclusions are based on the evidence with the highest confidence when considering evidence across study types and multiple outcomes. Confidence ratings are initially set based on key design features of the available studies for a given outcome (e.g., for experimental studies separately from observational studies). The studies with the highest confidence rating form the basis of the confidence conclusion for each evidence stream. As noted above, consistent results across studies with different design features increase confidence in the combined body of evidence and can result in an upgraded confidence rating moving forward to step 6. If the only available body of evidence receives a “very low confidence” rating, then conclusions for those outcomes will not move on to step 6.

After confidence conclusions are developed for a given outcome, conclusions for multiple outcomes are developed. The project-specific definition of an outcome and the grouping of biologically related outcomes used in this step follow the definitions developed *a priori* in the protocol; deviations are taken with care, justified, and documented. When outcomes are sufficiently biologically related that they may inform confidence on the overall health outcome, confidence conclusions may be developed in two steps. Each outcome would first be considered separately. Then, the related outcomes would be considered together and reevaluated for properties that relate to downgrading and upgrading the body of evidence.

### Step 6: Translate the Confidence Ratings into Level of Evidence for Health Effect

The level of evidence is assessed separately within the human, experimental animal, and—to the extent possible and necessary—other relevant data sets. The conclusions for the level of evidence for health effects reflect the overall confidence in the association between exposure to the substance and the outcome (effect or no effect; Figure 1, step 6). The strategy uses four terms to describe the level of evidence for health effects. These descriptors reflect both the confidence in the body of evidence for a given outcome and the direction of effect. Three descriptors used in step 6 (“high level of evidence,” “moderate level of evidence,” and “low level of evidence”) directly translate from the confidence-in-the-evidence ratings that exposure to the substance is associated with a heath effect, and a fourth designation (“evidence of no health effect”) indicates confidence that the substance is not associated with a health effect (for definitions of the level of evidence for health effects descriptors, see Supplemental Material, Table S3). Because of the inherent difficulty in proving a negative, the conclusion “evidence of no health effect” is reached only when there is high confidence in the body of evidence. In the context of evidence potentially supporting a conclusion of no health effect, a low or moderate level of evidence results in a conclusion of inadequate evidence to reach a conclusion.

Although the conclusions describe associations, a causal relationship is implied and the ratings describe the level of evidence for health effects in terms of confidence in the association or the estimate of effect determined from the body of evidence. Table 3 outlines how the Bradford Hill considerations on causality (Hill 1965) are related to the process of evaluating the confidence in the body of evidence and then integrating the evidence (similar to GRADE approach as described by Schünemann et al. 2011).

**Table 3:** Aspects of the Hill considerations on causality within the OHAT Approach.

Hill consideration	Relationship to the OHAT Approach
Strength	Considered in upgrading the confidence rating for the body of evidence for large magnitude of effect and downgrading the confidence rating for imprecision.
Consistency	Considered in upgrading the confidence rating for the body of evidence for consistency across study types, across dissimilar populations, or across animal species; in integrating the body of evidence among human, animal, and other relevant data; and in downgrading the confidence rating for the body of evidence for unexplained inconsistency.
Temporality	Considered in initial confidence ratings by key features of study design; for example experimental studies have an initial rating of “high confidence” because of the increased confidence that the controlled exposure preceded outcome.
Biological gradient	Considered in upgrading the confidence rating for the body of evidence for evidence of a dose–response relationship.
Biological plausibility	Considered in examining nonmonotonic dose–response relationships and developing confidence rating conclusions across biologically related outcomes, particularly outcomes along a pathway to disease; considered in downgrading the confidence rating for the body of evidence for indirectness. Other relevant data that inform plausibility, such as physiologically based pharmacokinetic and mechanistic studies, are considered in integrating the body of evidence.
Experimental evidence	Considered in setting initial confidence ratings by key features of study design and in downgrading the confidence rating for risk of bias.

### Step 7: Integrate the Evidence to Develop Hazard Identification Conclusions

The highest level of evidence for a health effect from each of the evidence streams is combined in the final step of the evidence assessment process to determine the hazard identification conclusion. Hazard identification conclusions may be reached on individual outcomes (health effects) or groups of biologically related outcomes, as appropriate, based on the evaluation’s objectives and the available data. The rationale for such conclusions is documented as the evidence is combined within and across evidence streams, and the conclusions are clearly stated as to which outcomes are incorporated into each conclusion. The five hazard identification conclusion categories are:

Known to be a hazard to humans
Presumed to be a hazard to humans
Suspected to be a hazard to humans
Not classifiable as a hazard to humans
Not identified to be a hazard to humans.

In step 7, the evidence streams for human studies and nonhuman animal studies, which have remained separate through the previous steps, are integrated along with other relevant data. Hazard identification conclusions are reached by integrating the highest level-of-evidence conclusion for a health effect(s) from the human and the animal evidence streams. On an outcome basis, this approach applies to whether the data support a health effect conclusion or evidence of no health effect.

When the data support a health effect, the level-of-evidence conclusion for human data from step 6 (“high,” “moderate,” or “low”) is considered together with the level of evidence for nonhuman animal data to reach one of four hazard identification conclusions (Figure 1, step 7). If one evidence stream (either human or animal) has no studies, then conclusions are based on the remaining evidence stream alone (which is equivalent to treating the missing evidence stream as “low”).

Any impact of other relevant data on the hazard identification conclusion derived by integrating the human and nonhuman animal streams is considered next (Figure 1, step 7). Other relevant data could include, but are not limited to, mechanistic data, *in vitro* data, or data based on upstream indicators of a health effect. Mechanistic data or another type of other relevant data is not required to reach a final hazard identification conclusion.

If other relevant data provide strong support for biological plausibility of the relationship between exposure and the health effect, the hazard identification conclusion may be upgraded (Figure 1, step 7, black “up” arrows) from that initially derived by considering the human and nonhuman animal evidence together. It is envisioned that strong evidence for a relevant biological process from mechanistic or *in vitro* data could result in a conclusion of “suspected” in the absence of human epidemiology or experimental animal data.

If other relevant data provide strong opposition for biological plausibility of the relationship between exposure and the health effect, the hazard identification conclusion may be downgraded (Figure 1, step 7, gray “down” arrows).

When the data provide evidence of no health effect, the level-of-evidence conclusion for human data from step 6 is considered together with the level-of-evidence for health effects conclusion for nonhuman animal data. Again, any impact of other relevant data on the hazard identification conclusion is considered.

If the human level-of-evidence conclusion of no health effect is supported by animal evidence of no health effect, the hazard identification conclusion is “not identified.”

The outcome of the evaluation includes any hazard identification conclusions reached or data needs identified, along with a detailed rationale outlining how human, animal, and other relevant data contributed to the conclusions. Draft OHAT evaluations undergo peer review and public comment as part of the overall process for finalization and publication (NTP 2013g).

## Discussion

Aspects of systematic-review methodology designed to increase objectivity and transparency may add to the time and investment required to develop literature-based evaluations, and the NTP is mindful of these concerns. In applying the OHAT Approach to case studies (NTP 2013a, 2013f), the NTP found that steps 2–4 were the most time intensive: selecting studies, extracting data, and assessing the quality of individual studies. Although not formally part of the systematic-review process, data management resources were used to increase transparency and efficiency in developing the case studies so that time invested in the early steps was recouped in later steps by entering study information into a database. Summary tables and graphics were readily made from the database to facilitate decision making in steps 6 and 7 when evaluating confidence in a body of studies and integrating evidence streams to develop conclusions. The value of these efficiencies and further development of these web-based systems for data display, data management, and data sharing cannot be understated.

## Conclusions

Applying systematic-review methodologies to environmental health questions is gaining a critical mass (EFSA 2010; NRC 2013a, 2013b; Woodruff and Sutton 2011). The OHAT Approach provides a practical method for applying the principles of systematic review to address environmental health questions. Moving forward, OHAT will apply this framework in future evaluations (NTP 2014b). As evaluations are completed and practices in the field of systematic review evolve, OHAT may refine and amend its “evergreen” approach and post updates to the framework (NTP 2013d). The protocols and the data compiled as part of an evaluation (e.g., study-level health effects data and risk-of-bias assessment) will be publicly available following its completion to increase transparency and facilitate data sharing with government agencies, the scientific community, and the public. The scientifically rigorous and objective procedures that have been a hallmark of OHAT literature-based health assessments will be strengthened by implementation of the OHAT Approach for systematic review and evidence integration (NTP 2013b).

The application of the procedures of systematic review to environmental health questions has the potential to increase objectivity and transparency much like it has already done for clinical medicine. Developing evaluations with this approach can improve communication and clarity about how hazard identification conclusions are reached by documenting the source of the data considered, the methods of quality assessment used, and the scientific judgments made during evidence integration.

## Supplemental Material

(247 KB) PDF
