# Supplementary material for: Systematic Review and Evidence Integration for Literature-Based Environmental Health Science Assessments
Source: Environ Health Perspect. 2014 Apr 22;122(7):711–8. doi: 10.1289/ehp.1307972 (PMC4080517; doi:10.1289/ehp.1307972)
Supplement: (247 KB) PDF [file ehp.1307972.s001.pdf]

Environmental Health Perspectives doi:10.1289/ehp.1307972

Supplemental Material

Systematic Review and Evidence Integration for Literature-Based Environmental Health Science Assessments

Andrew A. Rooney, Abee L. Boyles, Mary S. Wolfe, John R. Bucher, and Kristina A. Thayer

Table of Contents **Page**

Process for developing the OHAT Approach **2**

Survey and exploration of published systematic-review methodology *2*

Engagement with the NTP Board of Scientific Counselors *3*

Engagement with NTP-agency partners *5*

Stakeholder outreach and communication *5*

Table S1. Advisors consulted during development of OHAT Approach **8**

Table S2. Study design features for initial confidence rating in body of evidence schematic (step 5 of the OHAT Approach) **9**

Table S3. Level of evidence for health effects descriptors **12**

References **13**

Process for developing the OHAT Approach

We used a multi-pronged strategy to develop the OHAT Approach that included consultation with technical experts in systematic review and human health assessment, our scientific advisory committees, other agencies or programs that conduct literature-based health assessments, and through public comment by stakeholders. The method was refined by considering expert and public comments, including by reviewers of this manuscript, and through consideration of its application to case studies.

Survey and exploration of published systematic-review methodology

In 2011 we began exploring systematic-review methodology as a means to enhance transparency and clarity for how literature-based health assessments carried out by the NTP Office of Health Assessment and Translation (OHAT) are conducted and how the conclusions are reached. During 2012, we organized and hosted a series of webinars to survey and explore the systematic-review process through expert consultation on existing methods (e.g., AHRQ, CAMARADES Group, Cochrane, GRADE). These methodologies from the field of clinical medicine are most developed for assessing data from human clinical trials to reach health care recommendations, and therefore typically consider small datasets of similar study design in developing conclusions. Given the greater breadth of data relevant to environmental health questions (e.g., observational human, experimental animal, and *in-vitro* data), the webinars also focused on evaluating the potential application and adaptation of published methods to the evidence streams common to environmental health sciences. We also shared information and interacted with other groups working on applying systematic-review methodology to environmental health assessments (e.g., Navigation Group at University of California San Francisco, U.S. Environmental Protection Agency [EPA], and Agency for Toxic Substances and Disease Registry [ATSDR]).

Engagement with the NTP Board of Scientific Counselors

The NTP Board of Scientific Counselors (BSC), our primary external advisory group, played a key role in reviewing and critiquing our plans and draft approaches for implementing systematic review into NTP literature-based health assessments. There were multiple public meetings of the BSC at which the development of a new systematic-review approach was discussed, and these meetings also provided members of the public a forum to be informed or to comment. At the June 2012 BSC meeting, we first outlined plans to develop an approach for systematic review and evidence integration in OHAT literature-based health assessments (NTP 2012a). Subsequent to that initial public vetting, a working group of the BSC met in August 2012 to review an initial Draft NTP Approach that built on guidance from authoritative sources and technical input for carrying out systematic review. This initial draft approach laid out a detailed structure for carrying out the steps of a systematic review and for synthesizing data to reach hazard-identification conclusions.

The working group was composed of experts knowledgeable in systematic-review methodology as well as hazard assessment (Supplemental Material, Table S1). They were charged to evaluate the suitability and transparency of the proposed approach for reaching evidence assessment conclusions from a body of evidence (collection of studies) for noncancer health effects. The working group chair presented the outcome of their deliberations to the BSC at its meeting in December 2012, and the BSC unanimously accepted the working group's report (NTP 2012b). Overall, the working group commended the NTP for taking proactive steps to move forward the state of the science for hazard assessment. Two primary recommendations were (1) that the NTP approach document should not be a treatise as written in the initial draft, and instead, should

provide a framework that defines the steps and structure for the systematic review, and (2) that a protocol should be prepared for each project with the specific details for that evaluation.

We carefully considered the working group's feedback and the initial Draft NTP Approach was revised based on all comments, including the restructuring of the approach as a framework document with project-specific details moved to protocols developed for each evaluation. At the BSC meeting, we presented our response to the working group's report along with a Revised Draft NTP Approach, which laid out our framework for systematic review and evidence integration and identified the protocol as the vehicle to detail a specific evaluation. We also proposed to carryout case studies to develop two project-specific protocols as recommended, and to apply the approach to test cases as a means of identifying needed refinement or revision to the approach.

In response to public input at the December BSC meeting, in February 2013, the NTP released for public comment the framework document–*Draft OHAT Approach for Systematic Review and Evidence Integration for Literature-based Health Assessments – February 2013* (previously called "NTP Approach," now "OHAT Approach") (NTP 2013a). This was followed in April by release of the draft protocols for two systematic-review case studies: (1) the association of bisphenol A exposure and obesity and (2) the association of perfluorooctanoic acid and perfluorooctane sulfonate exposure and immunotoxicity (NTP 2013b, c).

In June 2013 we shared the draft OHAT Approach, draft protocols for the case studies, and public comments with the BSC at a public meeting. We presented how the draft addressed major technical or scientific issues in those comments including evaluation of study quality, the method for determining the initial rating for assessing confidence in the body of evidence, the impact of

excluding studies based on quality, and use of other relevant data (*in-vitro* and mechanistic data, etc.). The BSC responded favorably to the Draft OHAT Approach, its consideration of public input, and our proposed means for resolving remaining issues. Their input supported us moving forward with finalizing the approach document (NTP 2013d).

Engagement with NTP-agency partners

Throughout development of the OHAT Approach, we periodically shared drafts and communicated our strategy with NTP-agency partners through webinars and briefings to the NTP Executive Committee (NTP 2013e). Several agencies (EPA and ATSDR) were actively considering modifications to their literature-based evaluation processes, and therefore discussions on our draft approach included potential for harmonizing data extraction, developing templates, and sharing data files to the extent possible within the differing agency mandates. We also readily accepted invitations from our partners to share details of the approach at their events (e.g., EPA's 2013 Systematic Review Workshop). It was important to understand any potential concerns with our proposal to use systematic-review methodology to assess the scientific evidence and reach hazard conclusions since many of these agencies use OHAT health hazard assessments in their evaluations.

Stakeholder outreach and communication

Our strategy to develop the Draft OHAT Approach included several means for stakeholder outreach and communication. To facilitate sharing of information and obtaining public input during development of the approach, we established a webpage (http://ntp.niehs.nih.gov/go/38673) on the NTP website that catalogs documents and public

activities (NTP 2013f) and communicates information about public events through NTP Listserv and Federal Register notices.

Informing the toxicology and environmental health sciences communities about the Draft OHAT Approach, which was released for public comment in February 2013 (see above), and gaining their input were important parts of our strategy. The NTP employed a public-comment period on the Draft OHAT Approach of approximately 3 months during which time we undertook several activities to raise awareness. For example, we actively pursued opportunities to publicly discuss the methods including presentations on the approach at the NIEHS National Advisory Environmental Health Sciences Council in February 2013, an exhibitor-hosted session at the Society of Toxicology meeting on March 12, 2013, and in April 2013 published a commentary in *Environmental Health Perspectives* on implementation of systematic review at the NTP (Birnbaum et al. 2013).

We held several public, informational webinars to gain additional input on targeted issues or share information about the Draft OHAT Approach and answer questions. In March 2013 we hosted a public, informational webinar on the assessment of data quality in animal studies that focused on methodological issues related to our implementation of systematic review (NTP 2013g); over 90 individuals registered for the event. We also held a public webinar on April 23, 2013, to provide an overview of the Draft OHAT Approach's framework, describe the contents of the case-study protocols, and respond to questions from the public on any of the documents (NTP 2013h). The format included presentations by NTP staff and time for responding to attendees' questions. This 4-hour event had over 100 attendees including national and international participation (Canada, Great Britain, and Germany) from academia, industry, non-government organizations, professional societies, and state and federal government agencies.

Finally, as we worked toward finalizing the OHAT Approach, we sought to provide additional clarity on some issues in the public comments and provide a further opportunity for public input. On September 26, 2013, we held a public, informational webinar to address specific topics and themes in the comments and discuss our progress on the case studies (NTP 2013i). The topics included evaluating study quality and utility, determining the initial confidence rating in the body of evidence by study design features, and integrating evidence to reach conclusions. The case studies are currently in progress, and at the webinar we noted that once they are completed the NTP would hold a "lesson learned" webinar on the OHAT Approach using examples from the case studies. Similar to the April 2013 webinar, there was broad representation of stakeholder groups among the approximately 80 national and international attendees, and the format included presentation by NTP on each topic followed by time for attendees' questions.

The NTP's efforts toward application of systematic-review methodology to OHAT's literature-based hazard evaluations engendered interest by two National Academy of Science committees charged with reviewing approaches used by EPA for Integrated Risk Information System (IRIS) assessments. In addition, this work has sparked interest by several international groups that carry out health hazard assessments (e.g., European Food Safety Authority and Karolinska Institute for Environmental Medicine) and fostered additional opportunities for discussion on harmonization of tools and data sharing. The responses by these authoritative members of the risk assessment community to the OHAT Approach have been very favorable. For example, the interim report of the NAS Committee on Inorganic Arsenic supports the use of systematic review procedures for the EPA's IRIS assessment of arsenic and cites the OHAT Approach to incorporate systematic review procedures into literature-based environmental health assessments (NRC 2013).

Table S1. Advisors consulted during development of OHAT Approach.

**Technical advisors**	
Gordon Guyatt	Co-chair, Grading of Recommendations Assessment, Development and Evaluation (GRADE) Working Group, McMaster University
Malcolm Macleod	CAMARADES Centre, University of Edinburgh
Karen Robinson	Co-Director, Evidence-Based Practice Center, The Johns Hopkins Bloomberg School of Public Health
Holger Schünemann	Co-chair, GRADE Working Group, McMaster University
Tracey Woodruff	Director, Program on Reproductive Health and the Environment, University of California San Francisco
**NTP Board of Scientific Counselors Working Group**	
Lynn Goldman, Chair	Dean, School of Public Health and Health Services, George Washington University
Reeder Sams, Vice-chair	Acting Deputy Director, National Center for Environmental Assessment/Research Triangle Park Division, US Environmental Protection Agency
Lisa Bero	Director, San Francisco Branch, United States Cochrane Center at University of California San Francisco
Edward Carney	Senior Science Leader, Mammalian Toxicology, Dow Chemical Company
David Dorman	Professor, North Carolina State University
Elaine Faustman	Director, Institute for Risk Analysis and Risk Communication, University of Washington
Dale Hattis	Research Professor, George Perkins Marsh Institute, Clark University
Malcolm Macleod	CAMARADES Centre, University of Edinburgh
Tracey Woodruff	Director, Program on Reproductive Health and the Environment, University of California San Francisco
Lauren Zeise	Chief, Reproductive and Cancer Hazard Assessment Branch, Office of Environmental Health Hazard Assessment, California Environmental Protection Agency

OHAT sought advice during development of the OHAT Approach through consultation with technical experts in systematic review and human health assessments as well as scientific advisory groups and the public. The table lists technical advisors consulted on systematic-review methodologies in a series of NTP webinars in spring 2012 and the NTP Board of Scientific Counselors working group that reviewed an early draft of the OHAT Approach.

Table S2. Study design features for initial confidence rating in body of evidence schematic (step 5 of the OHAT Approach).

**Study design**	**Controlled exposure**	**Exposure prior to outcome**	**Individual outcome data**	**Comparison group used**
Human controlled trial[a]	likely	likely	likely	likely
Experimental animal	likely	likely	likely	likely
Cohort	unlikely	may or may not	likely	likely
Case-control	unlikely	may or may not	likely	likely
Cross-sectional[b]	unlikely	unlikely	likely	likely
Ecologic	unlikely	may or may not	may or may not	likely
Case series/report	unlikely	may or may not	likely	unlikely

[a]Human controlled trial study design used here refers to studies in humans with a controlled exposure including randomized controlled trials and non-randomized experimental studies.

[b]Cross-sectional study design used here refers to population surveys with individual data (e.g., NHANES) distinct from population surveys with aggregate data on participants (i.e., ecologic studies).

Study-design labels can distinguish between the relative strengths of study designs, but they are imprecise and often include a mix of design features that impact the ability of a study to address causality. Instead, four key study-design features can be used to differentiate the ability of the study to address causality as reflected in the confidence that exposure preceded and was associated with the outcome. Table S2 illustrates whether or not the four features are "likely" present, "may or may not" be present, or "unlikely" to be present in typical studies for each of the study designs listed.

In the OHAT Approach, the presence or absence of these four features is assessed on an outcome-specific basis to set the initial confidence rating for each study. "Controlled exposure" of subjects to the substance is the factor that distinguishes experimental studies from observational studies, and the experimental study design will also typically include the other three key features in both human and animal studies. The key feature that distinguishes between the relative strengths of observational epidemiologic study designs is "exposure prior to outcome," (i.e., the exposure assessment represents exposures that occurred prior to the development of the outcome). In these cases, it is unlikely that an association could be the result of reverse causation−where the outcome contributes to the exposure. Prospective cohort studies usually have three key study design features; however, when the exposures and outcomes are assessed at the start of a prospective study, these results will only have two key features and more closely resemble a cross-sectional study.

Studies without individual-level information on outcomes and other covariates cannot control for additional confounding variables and may lead to inappropriate inferences or an "ecologic fallacy." This limitation is captured with the third key feature "individual outcome data." An ecologic study can refer to exposures assessed via aggregate data (air pollution by zip code of

residence) with individual subject outcome information (which would have the third feature), or it could refer to exposures and outcomes assessed on aggregate data (trends in a city's air pollution and hospitalizations for asthma) and not have the third feature.

Without a comparison group there is limited ability to evaluate the association of an exposure and outcome. The fourth key feature "comparison group used" distinguishes case series and case reports from the other study designs because they typically lack a comparison group. Consideration of whether the comparison group used is appropriate is captured in the risk of bias assessment.

Table S3. Level of evidence for health effects descriptors.

**Evidence descriptors**	**Definition**
High level of evidence	There is high confidence in the body of evidence for an association between exposure to the substance and the health outcome(s).
Moderate level of evidence	There is moderate confidence in the body of evidence for an association between exposure to the substance and the health outcome(s).
Low level of evidence	There is low confidence in the body of evidence for an association between exposure to the substance and the health outcome(s), or no data are available.
Evidence of no health effect	There is high confidence in the body of evidence that exposure to the substance is not associated with the health outcome(s).
Inadequate evidence	There is insufficient evidence available to assess if the exposure to the substance is associated with the health outcome(s).

References

Birnbaum LS, Thayer KA, Bucher JR, Wolfe MS. 2013. Implementing systematic review at the National Toxicology Program. Environ Health Perspect 121:A108-109.

NRC (National Research Council). 2013. Critical Aspects of EPA's IRIS Assessment of Inorganic Arsenic - Interim Report. The National Academies Press: 1-117. Available: http://www.nap.edu/catalog.php?record_id=18594 [accessed 7 November 2013].

NTP (National Toxicology Program). 2012a. Board of Scientific Counselors June 21-22, 2012 meeting. Meeting materials. Available: http://ntp.niehs.nih.gov/go/9741 [accessed 16 June 2013].

NTP (National Toxicology Program). 2012b. Board of Scientific Counselors December 11, 2012 meeting. Meeting materials. Available: http://ntp.niehs.nih.gov/go/9741 [accessed 21 February 2013].

NTP (National Toxicology Program). 2013a. Draft OHAT Approach for Systematic Review and Evidence Integration for Literature-based Health Assessments – February 2013. RTP, NC: Office of Health Assessment and Translation. Available: http://ntp.niehs.nih.gov/go/38138 [accessed 10 March 2013].

NTP (National Toxicology Program). 2013b. Draft Protocol for Systematic Review to Evaluate the Evidence for an Association Between Perfluorooctanoic Acid (PFOA) or Perfluorooctane Sulfonate (PFOS) Exposure and Immunotoxicity. RTP, NC: Office of Health Assessment and Translation. Available: http://ntp.niehs.nih.gov/go/38673 [accessed 9 April 2013].

NTP (National Toxicology Program). 2013c. Draft Protocol for Systematic Review to Evaluate the Evidence for an Association Between Bisphenol A (BPA) and Obesity. RTP, NC: Office of Health Assessment and Translation. Available: http://ntp.niehs.nih.gov/go/38673 [accessed 9 April 2013].

NTP (National Toxicology Program). 2013d. Board of Scientific Counselors June 25, 2013 meeting. Meeting materials. Available: http://ntp.niehs.nih.gov/go/9741 [accessed 1 November 2013].

NTP (National Toxicology Program). 2013e. NTP Executive Committee. Available: http://ntp.niehs.nih.gov/go/163 [accessed 28 January 2014].

NTP (National Toxicology Program). 2013f. OHAT Implementation of Systematic Review. Available: http://ntp.niehs.nih.gov/go/38673 [accessed 16 June 2013].

NTP (National Toxicology Program). 2013g. Webinar on the assessment of data quality in animal studies. March 20, 2013. Available: http://ntp.niehs.nih.gov/go/38752 [accessed 7 April 2013].

NTP (National Toxicology Program). 2013h. Informational meeting on the draft OHAT Approach for Systematic Review and Evidence Integration for Literature-based Health Assessments. April 23, 2013. Available: http://ntp.niehs.nih.gov/go/38751 [accessed 28 January 2014].

NTP (National Toxicology Program). 2013i. Webinar on OHAT Approach for Systematic Review. September 26, 2013. Available: http://ntp.niehs.nih.gov/go/40490 [accessed 28 January 2014].
